# Supplementary figures and images for: Consequences of PDGFRα+ fibroblast reduction in adult murine hearts
Source: eLife. 2022 Sep 23;11:e69854. doi: 10.7554/eLife.69854 (PMC9576271; doi:10.7554/eLife.69854)

Figure 1-source data 1: full unedited western blots.

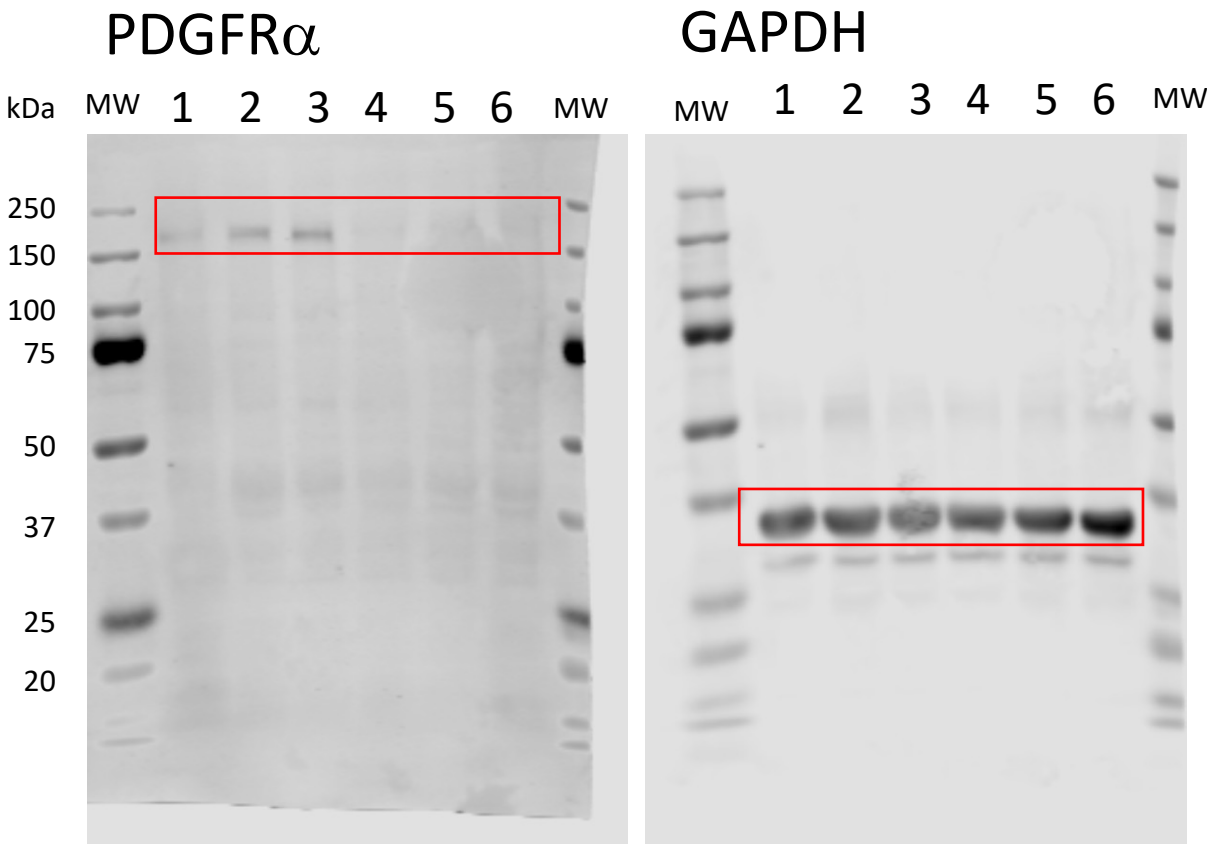

Lanes 1-3 control  
Lanes 4-6 ablated

Supplement: Figure 1—source data 1. [file elife-69854-fig1-data1.zip › Figure 1-source data 1.pdf]

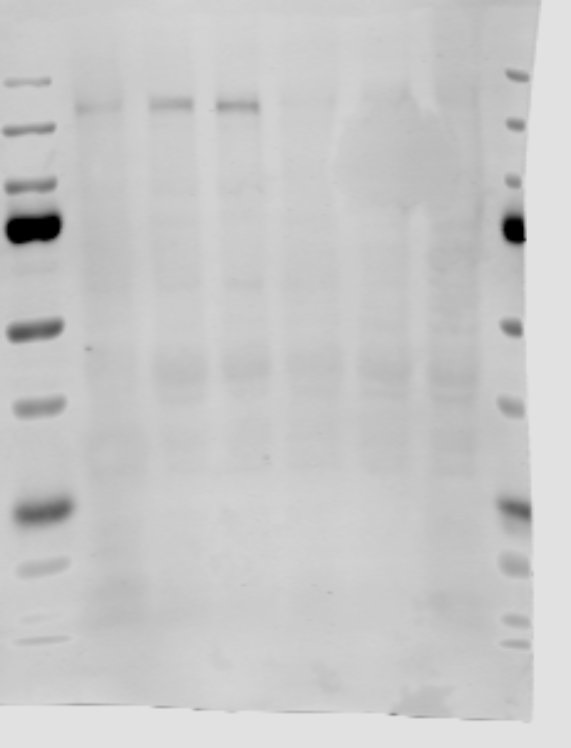

Supplement: Figure 1—source data 1. [file elife-69854-fig1-data1.zip › Figure 1G_PDGFRa.tif]

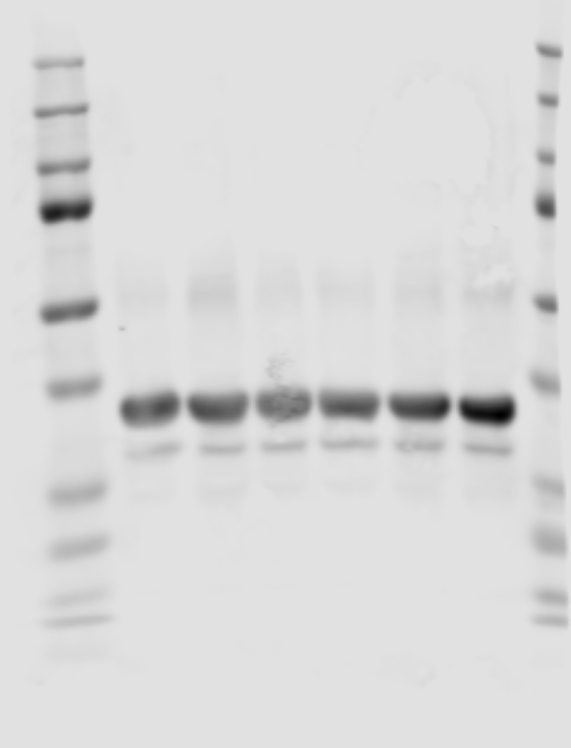

Supplement: Figure 1—source data 1. [file elife-69854-fig1-data1.zip › Figure 1G_PDGFRa_GAPDH.tif]

Figure 2-source data 1: full unedited western blots.

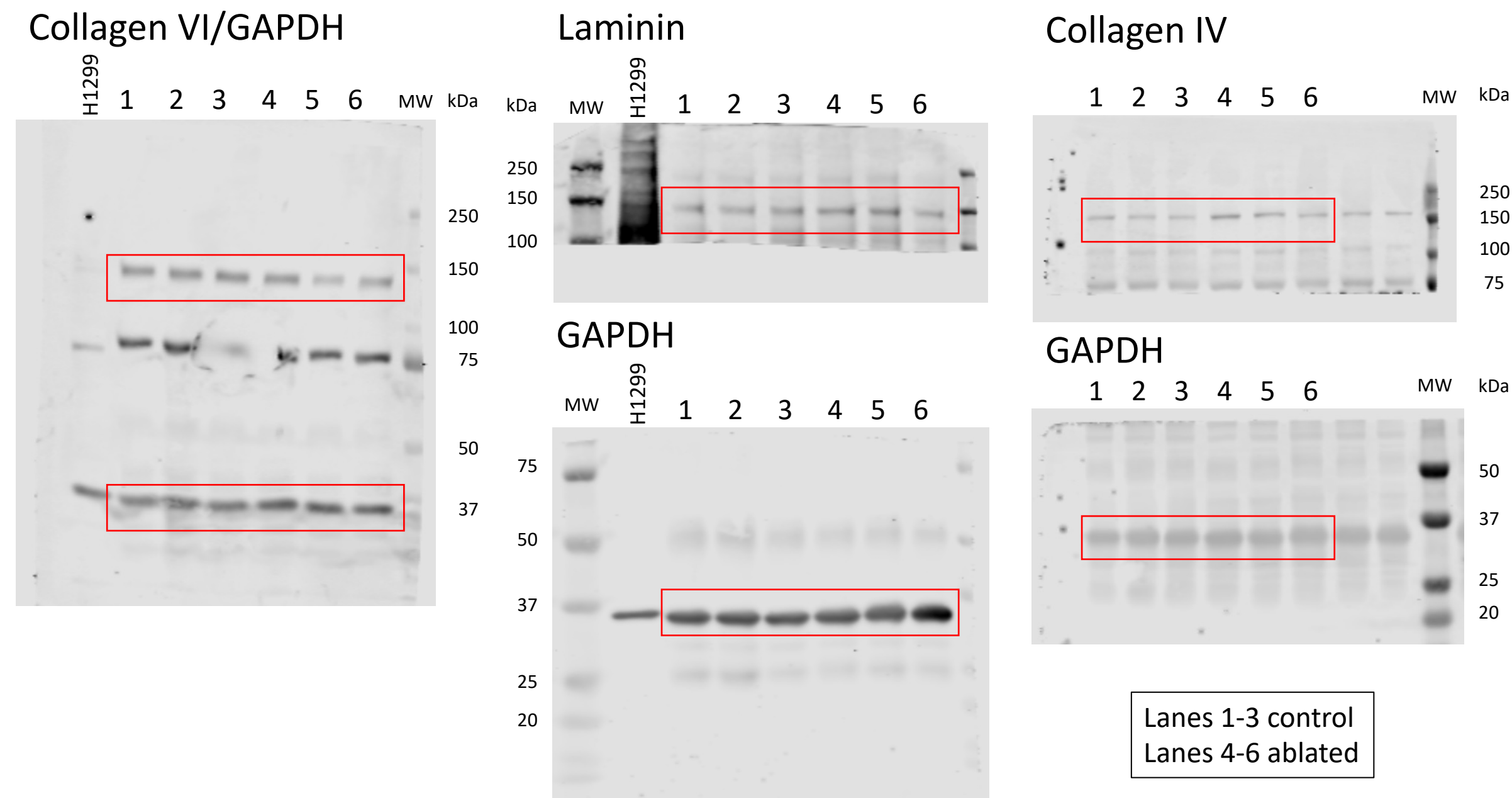

Supplement: Figure 2—source data 1. [file elife-69854-fig2-data1.zip › Figure 2-source data 1.pdf]

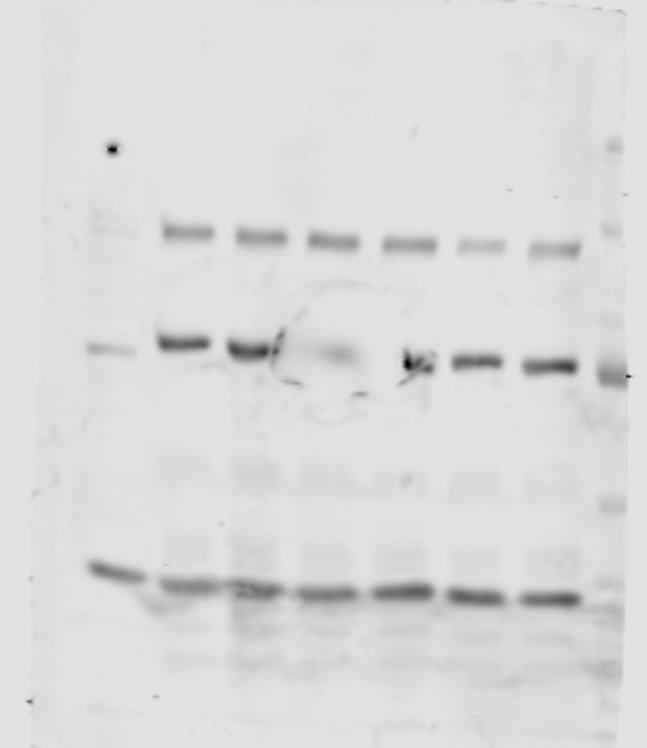

Supplement: Figure 2—source data 1. [file elife-69854-fig2-data1.zip › Figure 2L_collagen VI_GAPDH.tif]

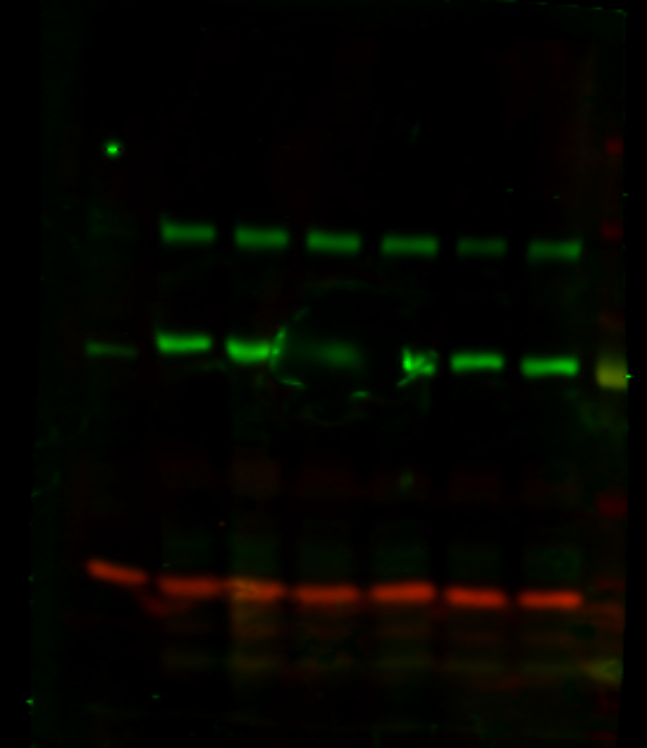

Supplement: Figure 2—source data 1. [file elife-69854-fig2-data1.zip › Figure 2L_collagen VI_GAPDH_700_800.tif]

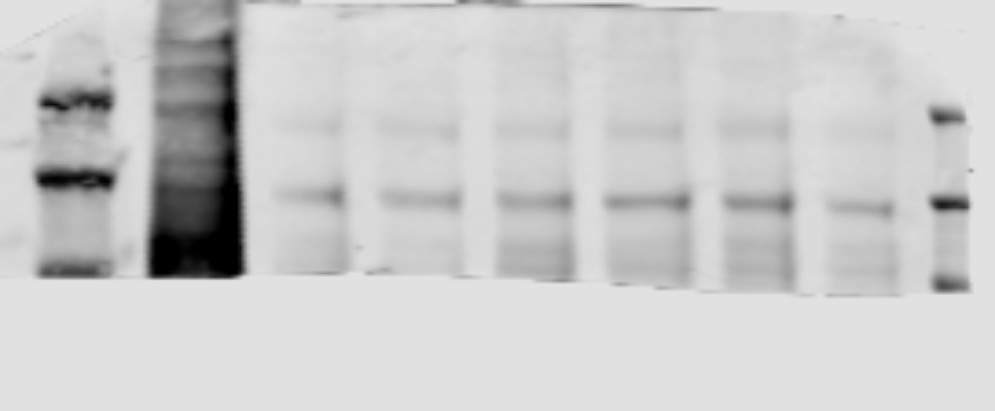

Supplement: Figure 2—source data 1. [file elife-69854-fig2-data1.zip › Figure 2L_laminin.tif]

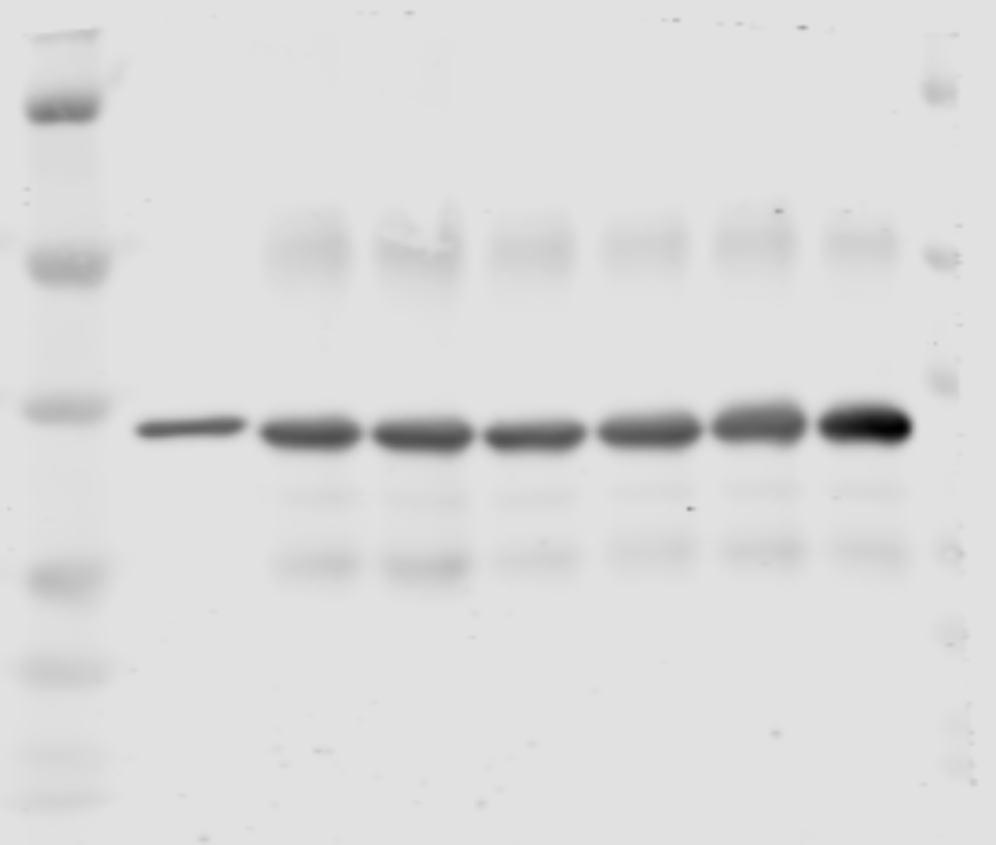

Supplement: Figure 2—source data 1. [file elife-69854-fig2-data1.zip › Figure 2L_laminin_GAPDH.tif]

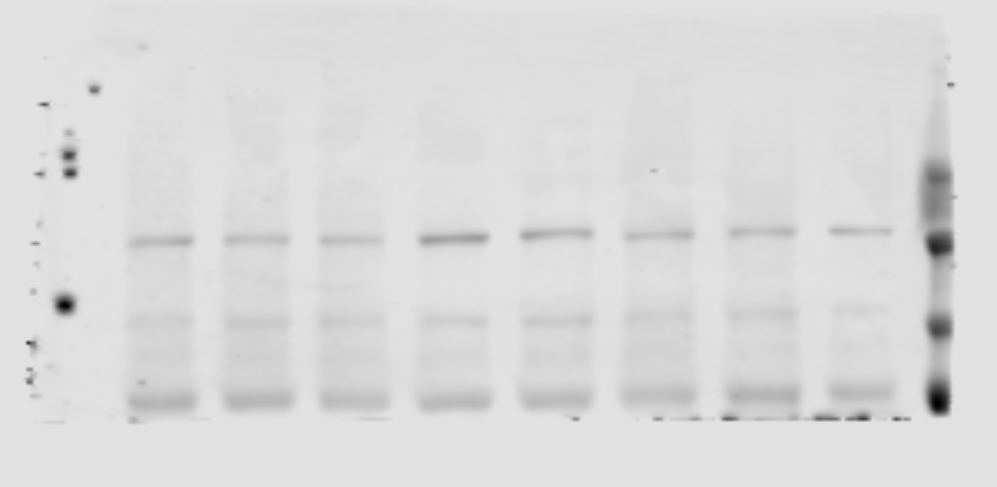

Supplement: Figure 2—source data 1. [file elife-69854-fig2-data1.zip › Figure 2L_collagen IV.tif]

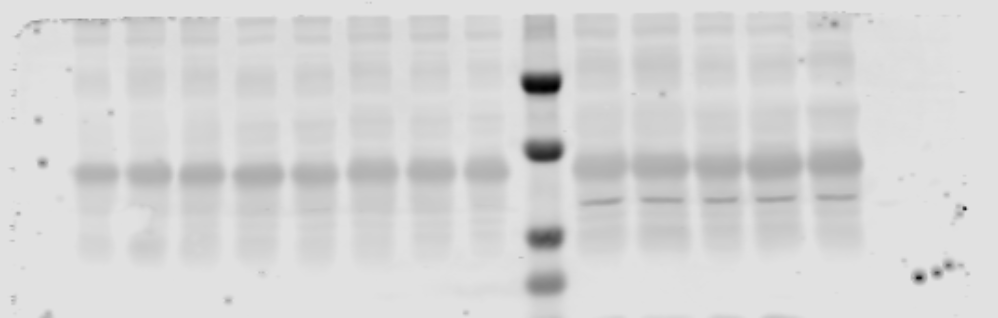

Supplement: Figure 2—source data 1. [file elife-69854-fig2-data1.zip › Figure 2L_collagen IV_GAPDH.tif]

Figure 3 -source data 1: full unedited zelatin zymogram

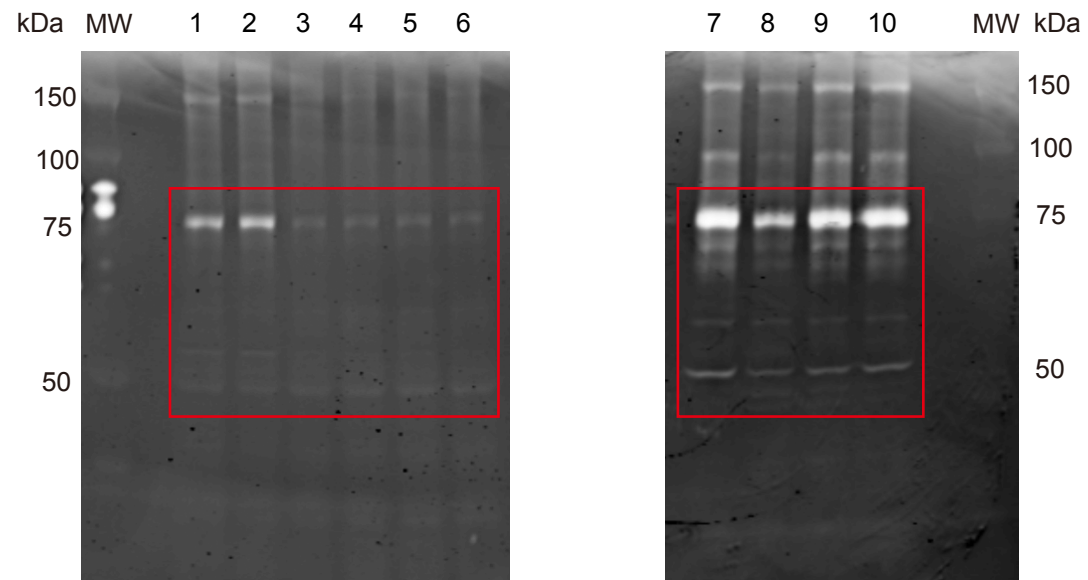

Lane 1-2, 7-8 control  
Lane 3-6, 9-10 ablated

Supplement: Figure 3—source data 1. [file elife-69854-fig3-data1.zip › R1_Figure 3_source data 1/R1_Figure 3- source data 1.pdf]

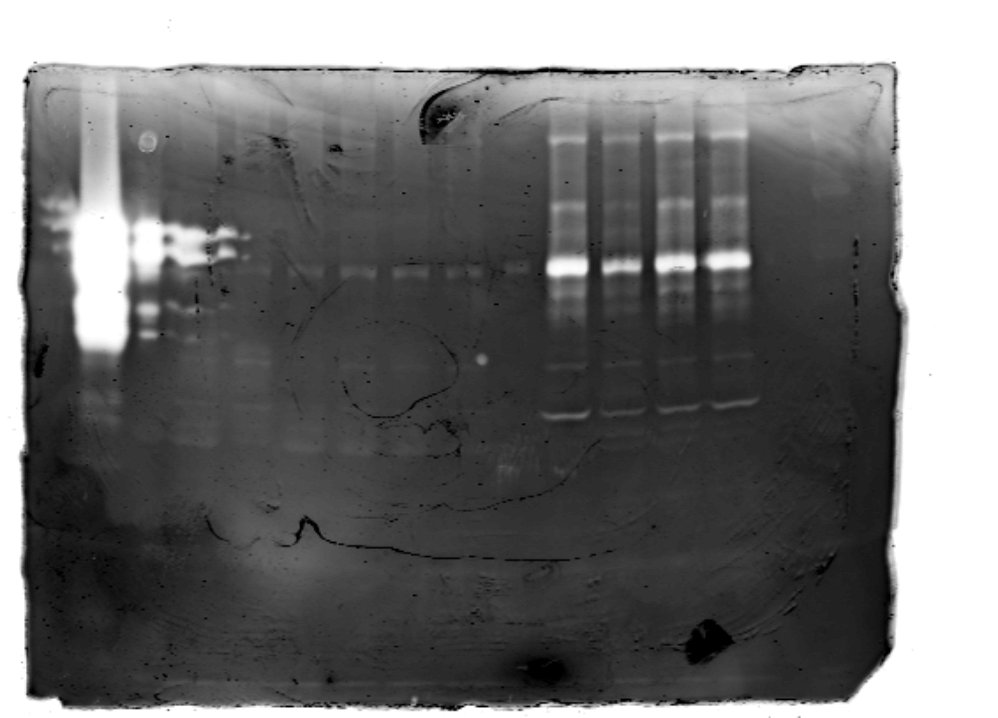

Supplement: Figure 3—source data 1. [file elife-69854-fig3-data1.zip › R1_Figure 3_source data 1/uneditted_source_MI3.tif]

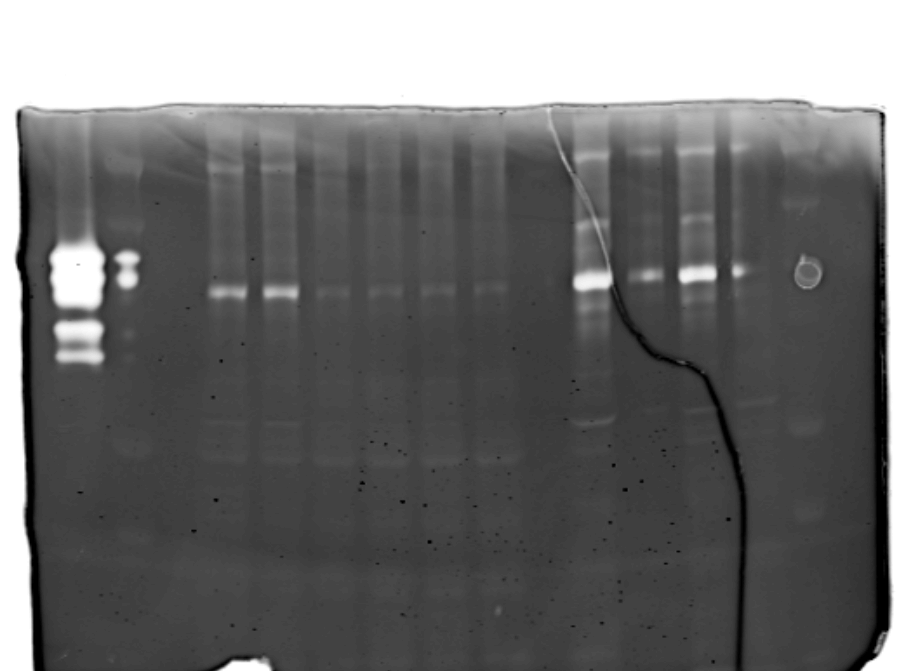

Supplement: Figure 3—source data 1. [file elife-69854-fig3-data1.zip › R1_Figure 3_source data 1/uneditted_source_baseline.tif]
