# Supplementary material for: Consequences of PDGFRα+ fibroblast reduction in adult murine hearts
Source: eLife. 2022 Sep 23;11:e69854. doi: 10.7554/eLife.69854 (PMC9576271; doi:10.7554/eLife.69854)
Supplement: Supplementary file 1. — (a) PDGFRβ+ population in Col1a1-GFP+ cells counted by flow cytometry. (b) Metabolic blood panel. (c) Potential proteolytically cleaved (internal) peptides with statistically significant higher abundance in control hearts. (d) Potential proteolytically cleaved peptides with statistically significant higher abundance in fibroblast-ablated hearts. Eghbali et al., 1988 (e) Efficiency of Pdgfra deletion determined for each experiment (expressed as the percent of cells deleted). (f) Cell-specific reagents for tissue/cell staining. (g) Primers used for qRT-PCR. [file elife-69854-supp1.docx]

**Supplementary file 1a. PDGFRβ^+^ population in *Col1a1*-GFP+ cells counted by flow cytometry.**

| **Group** | **Period post induction (wks)** | **PDGFRb^+^/ColG^+^**  **in CD31^-^/45^-^ population (%)** | **Mean**  **(± SD)** | **PDGFRb^+^/ColG^+^ (count / mg tissue)** | **Mean**  **(± SD)** |
| --- | --- | --- | --- | --- | --- |
| control | n.a. | 23.0 | 21.9  (±4.4) | 2053 | 2498  (±832) |
|  | n.a. | 16.3 |  | 1156 |  |
|  | n.a. | 24.0 |  | 2934 |  |
|  | n.a. | 20.0 |  | 3028 |  |
|  | n.a. | 19.1 |  | 2338 |  |
|  | n.a. | 29 |  | 3479 |  |
| ablated | 28 | 9.6 | 9.6  (±2.2) | 1370 | 962  (±353) |
|  | 28 | 9.2 |  | 1190 |  |
|  | 27 | 8.8 |  | 1138 |  |
|  | 29 | 6.9 |  | 574 |  |
|  | 27 | 9.7 |  | 1013 |  |
|  | 27 | 13.6 |  | 489 |  |
|  |  | p-value | *** 4.34 x 10^-3^ | p-value | *** 4.57 x 10^-3^ |

**Supplementary file 1b. Metabolic blood panel**

|  | Control | | | | | Ablated | | | | | | | Control  (Mean ± SD) | Ablated  (Mean ± SD) | *p-*value |
| --- | --- | --- | --- | --- | --- | --- | --- | --- | --- | --- | --- | --- | --- | --- | --- |
| Sodium  (mmol/L) | 148 | 148 | 149 | 150 | 150 | 146 | 146 | 147 | 151 | 147 | 146 | 146 | 149 ± 1.0 | 147 ± 1.8 | ns |
| Potassium  (mmol/L) | 5 | 6.3 | 5.4 | 5.4 | 5.4 | 6.2 | 5.3 | 5.7 | 5.7 | 6 | 5.3 | 6.3 | 5.5 ± 0.5 | 5.8 ± 0.4 | ns |
| Chloride  (mmol/L) | 114 | 119 | 116 | 114 | 116 | 118 | 117 | 113 | 120 | 117 | 114 | 113 | 116 ± 2.1 | 116 ± 2.7 | ns |
| Ionized Calcium  (mmol/L) | 1.28 | 1.25 | 1.29 | 1.35 | 1.3 | 1.28 | 1.31 | 1.27 | 1.32 | 1.27 | 1.29 | 1.37 | 1.30 ± 0.04 | 1.30 ± 0.03 | ns |
| Total CO2  (mmol/L) | 15 | 15 | 16 | 18 | 14 | 12 | 14 | 16 | 12 | 15 | 16 | 19 | 15.6 ± 1.5 | 14.9 ± 2.5 | ns |
| Glucose  (mg/dL) | 213 | 183 | 261 | 245 | 254 | 241 | 195 | 220 | 192 | 203 | 369 | 219 | 231 ± 33 | 234 ± 62 | ns |
| Blood Urea Nitrogen  (mg/dL) | 23 | 28 | 30 | 21 | 22 | 23 | 20 | 20 | 21 | 29 | 23 | 28 | 24.8 ± 4.0 | 23.4 ± 3.7 | ns |
| Creatinine  (mg/dL) | <0.2 | <0.2 | <0.2 | <0.2 | <0.2 | <0.2 | <0.2 | <0.2 | <0.2 | <0.2 | <0.2 | <0.2 | < 0.2 | < 0.2 | ns |
| Hematocrit  (% PCV) | 46 | 43 | 45 | 45 | 46 | 36 | 35 | 36 | 39 | 35 | 37 | 36 | 45 ± 1.2 | 36 ± 1.4 | <0.0001 |
| Hemoglobin  (g/dL) | 15.6 | 14.6 | 15.3 | 15.3 | 15.6 | 12.2 | 11.9 | 12.2 | 13.3 | 11.9 | 12.6 | 12.2 | 15.3 ± 0.4 | 12.3 ± 0.5 | <0.0001 |
| Anion Gap  (mmol/L, calculated) | 25 | 21 | 24 | 24 | 26 | 23 | 21 | 24 | 26 | 23 | 22 | 21 | 24 ± 1.9 | 22.9 ± 1.8 | ns |

Grey: female; white: male. Results are mean ± SD. Statistical significance was determined by unpaired t-test.

**Supplementary file 1c. Potential proteolytically cleaved (internal) peptides with statistically significant higher abundance in control hearts.** The annotated sequence shows at its N-terminal end the cleaved peptide bond which was deduced from the peptide sequence and the presence of an N-terminal label (.). The residue preceding the scissile bond (e.g., [A], corresponds to the P1 residue according to the nomenclature of Schechter and Berger (1). The position of the P1’ residue (which follows the scissile bond) is presented in column 4. The tryptic cut at the C-terminus is also shown, and invariably has a preceding Arg or Lys residue.

| **Accession number (UniProt)** | **Protein name** | **Annotated Sequence** | **P1’ AA Position** | **(log2): (Control / Ablated)** | **Adj. P-Value: (Control / Ablated)** |
| --- | --- | --- | --- | --- | --- |
| Q9CQ40 | 39S ribosomal protein L49, mitochondrial | [L].SQTQGPPDNPGFVESVDEYQFVER.[L] | 27 | 1.13 | 0.049233004 |
| Q99KI0 | Aconitate hydratase, mitochondrial | [R].YDLLEKNINIVR.[K] | 45 | 0.83 | 0.001630516 |
| P68134 | Actin, alpha skeletal muscle | [G].LVKAGFAGDDAPR.[A] | 18 | 1.65 | 6.23665E-06 |
| P60710 | Actin, cytoplasmic 1 | [W].DDMEKIWHHTFYNELR.[V] | 80 | 1.73 | 0.003692542 |
| P63268 | Actin, gamma-enteric smooth muscle | [NQ].EMATAASSSSLEKSYELPDGQVITIGNER.[F] | 226 | 1.04 | 4.9404E-05 |
| P63268 | Actin, gamma-enteric smooth muscle | [S].SSLEKSYELPDGQVITIGNER.[F]  [Y].VALDFENEMATAASSSSLEKSYELPDGQVITIGNER.[F]  [Y].ANNVLSGGTTMYPGIADR.[M]  [Q].GVMVGMGQKDSYVGDEAQSKR.[G]  [C].DIDIRKDLYANNVLSGGTTMYPGIADR.[M]  [S].SSSLEKSYELPDGQVITIGNER.[F]  [S].SLEKSYELPDGQVITIGNER.[F]  [S].LEKSYELPDGQVITIGNER.[F]  [G].FAGDDAPR.[A] | 234  221  297  42  288  233  235  236  21 | 0.88  0.83  1.14  0.85  2.22  0.87  0.71  0.75  0.94 | 0.00072102  0.001350125  0.001378523  0.001383431  0.003153518  0.003172322  0.007968922  0.012062644  0.012258745 |
| Q8K370 | Acyl-CoA dehydrogenase family member 10 | [H].STVAAASPSHEAKGGLVISPEGLSPAVR.[K] | 637 | 0.59 | 0.035970635 |
| P48962 | ADP/ATP translocase 1 | [C].FVYPLDFAR.[T] | 130 | 1.92 | 0.003905538 |
| P56480 | ATP synthase subunit beta, mitochondrial | [R].GVQKILQDYK.[S] | 423 | 0.58 | 0.042326665 |
| Q8BFZ3 | Beta-actin-like protein 2 | [Y].VAIQAVLSLYASGR.[T] | 134 | 1.53 | 0.045493203 |
| Q9CZU6 | Citrate synthase, mitochondrial | [H].ASASSTNLKDVLSNLIPKEQAR.[I] | 26 | 1.26 | 5.56119E-07 |
| Q68ED7 | CREB-regulated transcription coactivator 1 | [F].QSSGLDTSRTTR.[H] | 85 | 0.95 | 0.000204936 |
| Q9CZ13 | Cytochrome b-c1 complex subunit 1, mitochondrial | [T].ATFAQALQSVPETQVSILDNGLR.[V] | 36 | 0.84 | 0.003692542 |
| P19783 | Cytochrome c oxidase subunit 4 isoform 1, mitochondrial | [H].GSVVKSEDYAFPTYADRR.[D]  [A].HGSVVKSEDYAFPTYADRR.[D]  [G].SVVKSEDYAFPTYADRR.[D] | 25  24  26 | 1.45  1.31  1.27 | 1.28577E-09  1.11222E-06  0.000284487 |
| P12787 | Cytochrome c oxidase subunit 5A, mitochondrial | [H].GSHETDEEFDAR.[W] | 40 | 0.66 | 0.02951456 |
| Q9CPQ1 | Cytochrome c oxidase subunit 6C | [R].KAGIFQSAK.[-] | 68 | 1.49 | 1.28577E-09 |
| Q80XN0 | D-beta-hydroxybutyrate dehydrogenase, mitochondrial | [R].TTKSFLPLLR.[R] | 175 | 1.41 | 6.36704E-05 |
| Q9JKS4 | LIM domain-binding protein 3 | [Y].SAETLREMAQMYQMSLR.[G] | 200 | 0.7 | 0.030121229 |
| Q5SX40 | Myosin-1 | [V].AQWRTKYETDAIQR.[T]  [W].LPVYNAEVVAAYR.[G]  [L].PVYNAEVVAAYR.[G]  [P].VYNAEVVAAYR.[G] | 1372  131  132  133 | 1.82  1.2  1.13  0.99 | 0.003153518  0.008323708  0.010506035  0.041619738 |
| O08638 | Myosin-11 | [R].QKHTQAVEELTEQLEQFKR.[A] | 1199 | 0.59 | 0.042326665 |
| Q02566 | Myosin-6 | [E].QQVDDLEGSLEQEKKVR.[M]  [M].ADFGAAAQYLR.[K]  [Q].LEFNQIKAEIER.[K]  [I].LNPAAIPEGQFIDSR.[K]  [L].REQYEEEMEAKAELQR.[V]  [L].QSALEEAEASLEHEEGKILR.[A]  [Q].QVDDLEGSLEQEKKVR.[M]  [R].QLEEKEALISQLTR.[G]  [N].PAAIPEGQFIDSR.[K]  [Q].SALEEAEASLEHEEGKILR.[A]  [K].IQLEAKVKEMTER.[L]  [L].EEKEALISQLTR.[G]  [C].DLLREQYEEEMEAKAELQR.[V]  [Q].LEVEKLELQSALEEAEASLEHEEGKILR.[A]  [N].EIEDLMVDVER.[S] | 1029  7  1563  725  1346  1541  1030  1292  727  1542  913  1294  1343  1535  1424 | 1.08  1.04  0.9  0.89  0.76  0.88  1.02  0.74  0.94  0.72  0.67  0.74  1.71  1.02  0.76 | 5.39585E-05  0.000125178  0.001630516  0.004537537  0.006220997  0.006318734  0.009322742  0.010457201  0.010874433  0.013504196  0.015706071  0.023284843  0.027582409  0.033044627  0.039788277 |
| O70468 | Myosin-binding protein C, cardiac-type | [K].WLKDGVELTR.[E]  [C].STELFVKEPPVLITR.[S] | 482  440 | 0.68  1.23 | 0.01313844  0.016178058 |
| Q9D6J6 | NADH dehydrogenase [ubiquinone] flavoprotein 2, mitochondrial | [A].GGALFVHRDTPENNPDTPFDFTPENYKR.[I]  [A].GGALFVHR.[D] | 34  34 | 0.96  1.62 | 0.024431028  0.03218703 |
| P52503 | NADH dehydrogenase [ubiquinone] iron-sulfur protein 6, mitochondrial | [R].QKEVNENFAIDLIAQQPVNEVEHR.[I] | 52 | 0.88 | 0.001926476 |
| A2AAJ9 | Obscurin | [QR].MSKAAPVEWR.[K] | 4681 | 2.34 | 0.002949972 |
| O55126 | Protein NipSnap homolog 2 | [A].REDSWLKSLFVR.[K] | 36 | 1.78 | 1.73128E-12 |
| Q8K2B3 | Succinate dehydrogenase [ubiquinone] flavoprotein subunit, mitochondrial | [S].AKVSDAISTQYPVVDHEFDAVVVGAGGAGLR.[A] | 45 | 1.51 | 5.68118E-05 |
| P50752 | Troponin T, cardiac muscle | [F].MPNLVPPKIPDGERVDFDDIHR.[K] | 84 | 2.14 | 0.000261564 |
| P62984 | Ubiquitin-60S ribosomal protein L40 | [G].IIEPSLR.[Q] | 77 | 1.56 | 0.024771141 |
| P20152 | Vimentin | [Y].SSSPGGAYVTR.[S] | 54 | 1.22 | 7.1174E-07 |

**Supplementary file 1d. Potential proteolytically cleaved peptides with statistically significant higher abundance in fibroblast-ablated hearts.** The annotated sequence shows at its N-terminal end the cleaved peptide bond which was deduced from the peptide sequence and the presence of an N-terminal label (.). The residue preceding the scissile bond (e.g., [A], corresponds to the P1 residue according to the nomenclature of Schechter and Berger (1). The position of the P1’ residue (which follows the scissile bond) is presented in column 4.The tryptic cut at the C-terminus is also shown, and typically has a preceding Arg or Lys residue.

| **Accession number (UniProt)** | **Protein name** | **Annotated Sequence** | **P1’ AA Position** | **(log2): (Control ) / (Ablated)** | **Adj. P-Value: (Control ) / (Ablated)** |
| --- | --- | --- | --- | --- | --- |
| Q70FJ1 | A-kinase anchor protein 9 | [R].ELEQALLASAEPFP.[K] | 2115 | -2.03 | 0.006094 |
| Q5SX40 | Myosin-1 | [E].LEEEIEAER.[AT]  [Y].ETDAIQR.[T]  [L].LQAEIEELR.[A] | 1121  1379  1684 | -1.66  -1.15  -1.3 | 0.030995  0.001601  0.012259 |
| P13541 | Myosin-3 | [VI].EDLMVDVER.[AS] | 1426 | -1.35 | 0.037948 |
| Q5SX39 | Myosin-4 | [E].ATAAALR.[K] | 1190 | -1.29 | 0.000175 |
| Q02566 | Myosin-6 | [E].QDNLNDAEER.[C]  [Y].EESQSELESSQKEAR.[S]  [E].ESQSELESSQKEAR.[S]  [E].SQSELESSQKEAR.[S]  [L].QTSLDAETR.[S]  [ETA].SLDAETR.[RSA] | 897  1461  1462  1463  1598  1600 | -1.23  -1.64  -1.7  -1.39  -1.57  -1.21 | 0.014372  0.018789  0.000965  3.51E-05  2.69E-07  0.00071 |
| Q61941 | NAD(P) transhydrogenase, mitochondrial | [R].KTTVLAMDQVPR.[V] | 171 | -1.61 | 0.018789 |
| Q3V129 | Serine/threonine-protein kinase ULK4 | [D].PLPPIPKDSSFPK.[A] | 236 | -0.9 | 0.047896 |
| P58771 | Tropomyosin alpha-1 chain | [K].LKYKAISEELDHALNDMTSI. [-] | 265 | -2.64 | 4.71E-06 |

**Supplementary file 1e. Efficiency of PDGFRα deletion determined for each experiment (expressed as the percent of cells deleted).**

| **Figure** | **Experiment** | **% deletion** | **Method** |
| --- | --- | --- | --- |
| Figure 1A-B | tdTomato cell number quantification | 65 | tf |
|  |  | 72 | tf |
|  |  | 68 | tf |
|  |  | 64 | tf |
|  |  | 52 | tf |
| Figure 1C-E | GFP cell number quantification | 71 | gf |
|  |  | 87 | gf |
|  |  | 74 | gf |
|  |  | 75 | gf |
| Figure 1F | Flow cytometry | 70 | fc (PDGFRα^lin^) |
|  |  | 80 | fc (PDGFRα^lin^) |
|  |  | 42 | fc (PDGFRα^lin^) |
|  |  | 56 | fc (PDGFRα^lin^) |
|  |  | 56 | fc (PDGFRα^lin^) |
|  |  | 64 | fc (PDGFRα^lin^) |
| Figure 1F | Flow cytometry | 70 | fc (PDGFRα, MEFSK4) |
|  |  | 57 | fc (PDGFRα, MEFSK4) |
|  |  | 49 | fc (PDGFRα, MEFSK4) |
|  |  | 65 | fc (PDGFRα, MEFSK4) |
|  |  | 58 | fc (PDGFRα, MEFSK4) |
|  |  | 75 | fc (PDGFRα, MEFSK4) |
| Figure 1G-H | Western blot (2-4 months post-induction) | 79 | wb (PDGFRα) |
|  |  | 78 | wb (PDGFRα) |
|  |  | 93 | wb (PDGFRα) |
|  |  | 72 | wb (PDGFRα) |
|  |  | 79 | wb (PDGFRα) |
|  |  | 54 | wb (PDGFRα) |
| Figure 1I | Baseline qPCR | 61 | hq (PDGFRα) |
|  |  | 46 | hq (PDGFRα) |
|  |  | 72 | hq (PDGFRα) |
| Figure 1J | Fibroblast qPCR | 46 | hq (PDGFRα) |
|  |  | 29 | hq (PDGFRα) |
|  |  | 49 | hq (PDGFRα) |
|  |  | 46 | hq (PDGFRα) |
|  |  | 51 | hq (PDGFRα) |
| Figure 3A-D | PDGFRα, collagen I, laminin, collagen IV, collagen VI IHC (1 month post-induction) | 50 | ihc (PDGFRα) |
|  |  | 63 | ihc (PDGFRα) |
|  |  | 48 | ihc (PDGFRα) |
|  |  | 53 | ihc (PDGFRα) |
|  |  | 48 | ihc (PDGFRα) |
|  |  | all samples 50-70 | tf |
| Figure 3E-H, K | PDGFRα, collagen I, laminin, collagen IV, collagen VI IHC (7 months post-induction) | 60 | ihc (PDGFRα) |
|  |  | 57 | ihc (PDGFRα) |
|  |  | 47 | ihc (PDGFRα) |
|  |  | 74 | ihc (PDGFRα) |
|  |  | all samples 50-70 | tf |
| Figure 2A-C | Shotgun proteomics | 75 | kq (PDGFRα) |
|  |  | 74 | kq (PDGFRα) |
|  |  | 75 | kq (PDGFRα) |
|  |  | ~~33~~ | kq (PDGFRα) |
| Figure 2D | Gelatin zymogram | 73 | kq (PDGFRα) |
|  |  | 74 | kq (PDGFRα) |
|  |  | 63 | kq (PDGFRα) |
|  |  | 76 | kq (PDGFRα) |
|  |  | 71 | kq (PDGFRα) |
|  |  | 82 | kq (PDGFRα) |
| Figure 3I | Hydroxyproline assay | 91 | kq (PDGFRα) |
|  |  | 98 | kq (PDGFRα) |
|  |  | 60 | kq (PDGFRα) |
|  |  | 96 | kq (PDGFRα) |
|  |  | 83 | kq (PDGFRα) |
|  |  | 79 | kq (PDGFRα) |
|  |  | 81 | kq (PDGFRα) |
| Figure 3J | SEM | 76 | kq (PDGFRα) |
|  |  | 83 | kq (PDGFRα) |
|  |  | 90 | kq (PDGFRα) |
| Figure 3L | Western blot (7 months post-induction) | 57 | wb (PDGFRα) |
|  |  | 72 | wb (PDGFRα) |
|  |  | 76 | wb (PDGFRα) |
| Figure 4 | 28d AngII/PE histology | 69 | kq (PDGFRα) |
|  |  | 74 | kq (PDGFRα) |
|  |  | 68 | kq (PDGFRα) |
|  |  | all samples 70-80 | tf |
| Figure 5 | 14d AngII/PE microarray | 55 | hq (PDGFRα) |
|  |  | ~~12~~ | hq (PDGFRα) |
|  |  | 56 | hq (PDGFRα) |
|  |  | 61 | hq (PDGFRα) |
|  |  | 66 | hq (PDGFRα) |
|  |  | 49 | hq (PDGFRα) |
| Figure 7 | 10 weeks Post-MI | 86 | kq (PDGFRα) |
|  |  | 72 | kq (PDGFRα) |
|  |  | 80 | kq (PDGFRα) |
|  |  | 82 | kq (PDGFRα) |
|  |  | 65 | kq (PDGFRα) |
| Figure 1 Supplement 2G-H | PV loop analysis | 85 | kq (PDGFRα) |
|  |  | 47 | kq (PDGFRα) |
|  |  | 93 | kq (PDGFRα) |
|  |  | 93 | kq (PDGFRα) |
|  |  | 90 | kq (PDGFRα) |
| Figure 2 Supplement 2 | TAILS proteomics | 70 | kq (PDGFRα) |
|  |  | 88 | kq (PDGFRα) |
|  |  | 70 | kq (PDGFRα) |
|  |  | 88 | kq (PDGFRα) |
|  |  | ~~29~~ | kq (PDGFRα) |
|  |  | ~~56~~ | kq (PDGFRα) |

Abbreviations: tf, tomato fluorescence; gf, gfp fluorescence; wb, western blot; hq, qPCR from heart; kq, qPCR from kidney; fc, flow cytometry; strikethrough indicates sample excluded from study. Note: Perfusion techniques used for primary cardiomyocyte isolation resulted in poor RNA quality, thus no deletion efficiency could be evaluated for these samples.

**Supplementary file 1f. Cell-specific reagents for tissue/cell staining.**

| **Antibody/reagent** | **Clone** | **Application (conc. µg/ml)** | **Vendor (cat no.)** |
| --- | --- | --- | --- |
| Anti-collagen I | polyclonal | TS (10.0) | Genetex (GTX20292) |
| Anti-CD11b | M1/70 | FC (0.4) | BioLegend (101225) |
| Anti-CD140a | REA637 | FC (1.5) | Miltenyi (130-109-736) |
| Anti-CD140a | APA5 | FC (5.0) | Invitrogen (14-1401-81) |
| Anti-CD140b | APB5 | TS (5.0) | Invitrogen (14-1402-82) |
| Anti-CD140b | APB5 | FC (10.0) | Invitrogen (17-1402-82) |
| Anti-CD31 | REA784 | FC (3.0) | Miltenyi (130-111-356) |
| Anti-CD31 | 390 | FC (2.5) | BioLegend (102443) |
| Anti-CD45 | 30-F11 | FC (1.0) | BioLegend (103126) |
| Anti-CD45 | 30-F11 | FC (5.0) | Invitrogen (56-0451-82) |
| Anti-CD45 | REA737 | TS (0.6) | Miltenyi (130-110-657) |
| Anti-collagen IV | polyclonal | TS (1.0) | Millipore (AB748) |
| Anti-collagen IV | polyclonal | WB (1.0) | Thermo Fisher (PA5-104508) |
| Anti-collagen VI alpha 1 | SD83-03 | WB (1.0) | Novus biologicals (NBP2-67825) |
| Anti-GAPDH | 6C5 | WB (3.0) | Thermo Fisher (AM4300) |
| Anti-Gr-1 | RB6-8C5 | FC (0.2) | BioLegend (108411) |
| Anti-laminin | polyclonal | TS (5.0) | Sigma (L9393) |
| Anti-laminin | polyclonal | WB (1.0) | Thermo Fisher (PA1-16730) |
| Anti-Ly6G | 1A8-Ly6g | FC (0.2) | eBioscience (12-9668-80) |
| Anti-mEF-SK4 | mEF-SK4 | FC (7.5) | Miltenyi (130-120-166) |
| Anti-NG2 | polyclonal | TS (4.0) | Millipore (AB5320) |
| Anti-PDGFRα | polyclonal | TS (2.0) | R&D systems (AF1062) |
| Anti-PDGFRα | D1E1E | WB (0.023) | Cell signaling (3174) |
| Anti-vimentin | polyclonal | TS (10.0) | Abcam (ab45939) |
| Wheat germ agglutinin | NA | TS (5.0) | Vector Labs (B-1025) |
| TS: tissue staining; WB: Western blot; FC: Flow cytometry | | | |

**Supplementary file 1g. Primers used for qRT-PCR.**

| **Gene** | **Forward sequence (5'-3')** | **Reverse sequence (5'-3')** |
| --- | --- | --- |
| *Col1a1* | AATGGCACGGCTGTGTGCGA | AACGGGTCCCCTTGGGCCTT |
| *Col1a2* | GGCCCCCTGGTATGACTGGCT | CGCCACGGGGACCACGAATC |
| *Col5a1* | TGAGTCTGGTTTTCCCGAGGA | GCCCTGCTCATTGTAAATGGAGA |
| *Dcn* | AGTGTTAATTACATCGCAGCTTTG | AGAGTTGCCTTCATGATTATCTCA |
| *Dkk3* | CTCGGGGGTATTTTGCTGTGT | TCCTCCTGAGGGTAGTTGAGA |
| *Dpep1* | CCATCTGTGCTTCGGACTCAT | CCAAGGCAAGTCGTTGTGC |
| *Fbn1* | CATTCCTGTGGGGATGGATTC | TACGTGCAAGCACACCGATTT |
| *Frzb* | ACGGAGCGGATTTTCCTATGG | CACAACGGCGGTCACATCA |
| *Lum* | CTCTTGCCTTGGCATTAGTCG | GGGGGCAGTTACATTCTGGTG |
| *Mdk* | TGGAGCCGACTGCAAATACAA | GGCTTAGTCACGCGGATGG |
| *Pdgfra* | GTCGTTGACCTGCAGTGGA | CCAGCATGGTGATACCTTTGT |
| *Pdgfrl* | CGGACTTCTGTTGCTACACGA | TGGTTGGTTTGATCCTGTTTTCT |
| *Pdpn* | CACCTCAGCAACCTCAGAC | ACAGGGCAAGTTGGAAGC |
| *Sox9* | AGACCAGTACCCGCATCTGCACAA | TCTCTTCTCGCTCTCGTTCAGCA |
| *Tbx20* | CTGGATCAACACGGCCATATAAT | CTGGAAAGATGAACGTCCTGAA |
| *Tcf21* | GGCCAACGACAAGTACGAGA | GCTGTAGTTCCACACAAGCG |
| *Vim* | CCAACCTTTTCTTCCCTGAA | TGAGTGGGTGTCAACCAGAG |
| *Actb* | GTGACGTTGACATCCGTAAAGA | GCCGGACTCATCGTACTCC |
| *18s* | GTAACCCGTTGAACCCCATT | CCATCCAATCGGTAGTAGCG |

Reference:

1. Schechter I, and Berger A. On the size of the active site in proteases. I. Papain. 1967. *Biochemical and biophysical research communications.* 2012;425(3):497-502.
